# Incidence of antiepileptic drug use in Parkinson's disease

**DOI:** 10.1177/1877718X251343079

**Published:** 2025-05-23

**Authors:** Santeri Tuominen, Miia Tiihonen, Anne Paakinaho, Marjaana Koponen, Valtteri Kaasinen, Sirpa Hartikainen, Anna-Maija Tolppanen

**Affiliations:** 1 Faculty of Health sciences, School of Pharmacy, University of Eastern Finland, Kuopio, Finland; 2 Centre for Medicine Use and Safety, Faculty of Pharmacy and Pharmaceutical Sciences, Monash University, Parkville, Victoria, Australia; 3 Clinical Neurosciences, University of Turku, Turku, Finland; 4 Neurocenter, Turku University Hospital, Turku, Finland

**Keywords:** Parkinson's disease, antiepileptic, incidence, initiation, neuropathic pain

## Abstract

**Background:**

Antiepileptics are used to treat epilepsy but also, e.g., neuropathic pain, essential tremor and dystonia. It is not known whether they are more commonly used in persons with Parkinson's disease (PD).

**Objective:**

To assess the incidence of antiepileptic use in a nationwide cohort of persons with PD before and after the diagnosis and compared the findings to a matched cohort without PD.

**Methods:**

This register-based Finnish nationwide cohort included 18365 persons diagnosed with PD between 2001–2015. Incidence of antiepileptic initiations, from 10 years before until 10 years after the PD diagnosis, was compared to an age-, sex-, and region-matched cohort without PD.

**Results:**

Antiepileptics were more commonly initiated for persons with PD (29.3% of PD cohort and 15.2% of comparison cohort). Gabapentinoids were the most commonly initiated antiepileptics in both cohorts. A similar pattern in initiation rates was observed for both gabapentinoids and other antiepileptics, with increased incidence in the PD cohort approximately three years before the diagnosis and a significant peak around the time of PD diagnosis (the initiation rate at the time of PD diagnosis 3/100 and 1/100 person-years, for the PD and comparison cohorts, respectively). Clonazepam initiations were more common in the PD cohort (26.7% of initiations vs. 5.8% in the comparison cohort).

**Conclusions:**

The increase in antiepileptic initiation rates before the diagnosis of PD suggests that they might be used for prodromal motor or non-motor symptoms.

## Plain language summary

People with Parkinson's disease (PD) have different conditions such as pain and involuntary muscle spasms which can require different drug treatments. Antiepileptic drugs (AEDs) are a group of different drugs, commonly used for treating epilepsy but some of them may also be used to treat the aforementioned conditions, which may also occur already years before PD is diagnosed. It is not known how commonly AEDs are used in people with PD, although this would aid in understanding how certain non-motor symptoms of PD are treated over time. We studied the AED initiations in a Finnish nationwide study of 18365 people with PD and a matched comparison group without PD to assess how much more commonly AEDs are initiated in PD and when in relation to PD diagnosis these drugs are initiated. The study period began 10 years before PD diagnosis and ended 10 years after. AEDs were more commonly initiated in people with PD: 29.3% of people with PD and 15.2% without PD started AEDs during the study period. There was a stable increase in initiation rate in comparison group, reflecting ageing of the population. A steeper increase in PD cohort began three years before the PD diagnosis date and there was a clear peak in initiations around the PD diagnosis date. The increased incidence of these medications likely reflects treatment of PD-associated symptoms such as neuropathic pain and anxiety and underlines the clinical importance of early non-motor PD symptoms and their treatment needs.

## Introduction

During the progress of Parkinson's disease (PD) patients may experience various symptoms, such as neuropathic pain^1^ and dystonia,^2^ which are commonly treated with antiepileptics. In addition, persons with PD can have comorbidities, such as epilepsy or seizures,^3^ bipolar disorder^4^ and essential tremor,^5^ that may also be treated with antiepileptics.

Pain is common in PD, as approximately 55–70% of people diagnosed with PD suffer from musculoskeletal pain^6–8.^ PD-related pain can manifest already during the prodromal period,^9^ but the prevalence and severity of pain can increase during the disease progression.^10^ Although the motor symptoms are the hallmark of PD, non-motor symptoms and pain have a significant effect on the quality of life, and pain can be one of the most debilitating and severe of all non-motor symptoms, especially in early-stage PD.^11^

Previous studies have suggested bipolar disorder,^12^ epilepsy^10^ and specific antiepileptics as risk factors for PD.^13^ Still, there are no studies describing the initiation of antiepileptics in relation to PD diagnosis or comparing the initiation rate to persons without PD. Thus, very little is known on use of antiepileptics in this population, although this would aid us in understanding how certain symptoms of PD are treated over time.

We studied the initiation of antiepileptics in a Finnish nationwide study of persons diagnosed with PD between 2001–2015 from 10 years before the PD diagnosis until 10 years after the diagnosis and compared the rate to a matched cohort without PD.

## Methods

### The FINPARK study

This study was conducted within the register-based Finnish Parkinson's disease study (FINPARK), which includes 22,189 people diagnosed with PD, and a matched cohort of 148,009 people without PD. The matched cohort includes up to seven matched controls for each person with PD, matched on age (+/− 1 year), sex and hospital district on the PD diagnosis date (index date). The study population has been described in detail earlier.^14^ Briefly, the persons with incident eligibility for special reimbursement for PD medications in 1996–2015 were identified from the Special reimbursement register (N = 29,942). As these medications can be used also for other conditions than PD, those who did not have ICD-10 code for PD (G20) recorded in the Finnish Special Reimbursement Register (n = 1244) were excluded from the study. Further exclusions were those who were under 35 years old at the time of diagnosis (n = 53) and those who had specific exclusion diagnoses such as other neurodegenerative diagnoses within two years of PD diagnosis date (n = 6456) to minimize the risk of false diagnosis at the early stages of PD, when the diagnosis is most challenging.^15^ The proportion of excluded persons (25.9%) in this study is similar to that of estimated false diagnosis rate of PD.^16^ The same exclusion criteria were applied on the matched comparison cohort, with the addition of dementia in Parkinson's disease (ICD-10 code F02.3). To be eligible for reimbursement, the PD diagnosis needs to be confirmed in specialist settings, and the diagnostic statements are centrally reviewed and confirmed in the Social Insurance Institution of Finland. During the study period, the PD diagnosis criteria were consistent with the UK Brain Bank criteria.

This study was restricted to persons with index date between 2001 and 2015 (18,733 persons with PD and their 124,501 matched comparison persons) to ensure all included persons had at least five-year follow-up time before the PD diagnosis.

### Study design

**Figure 1.**
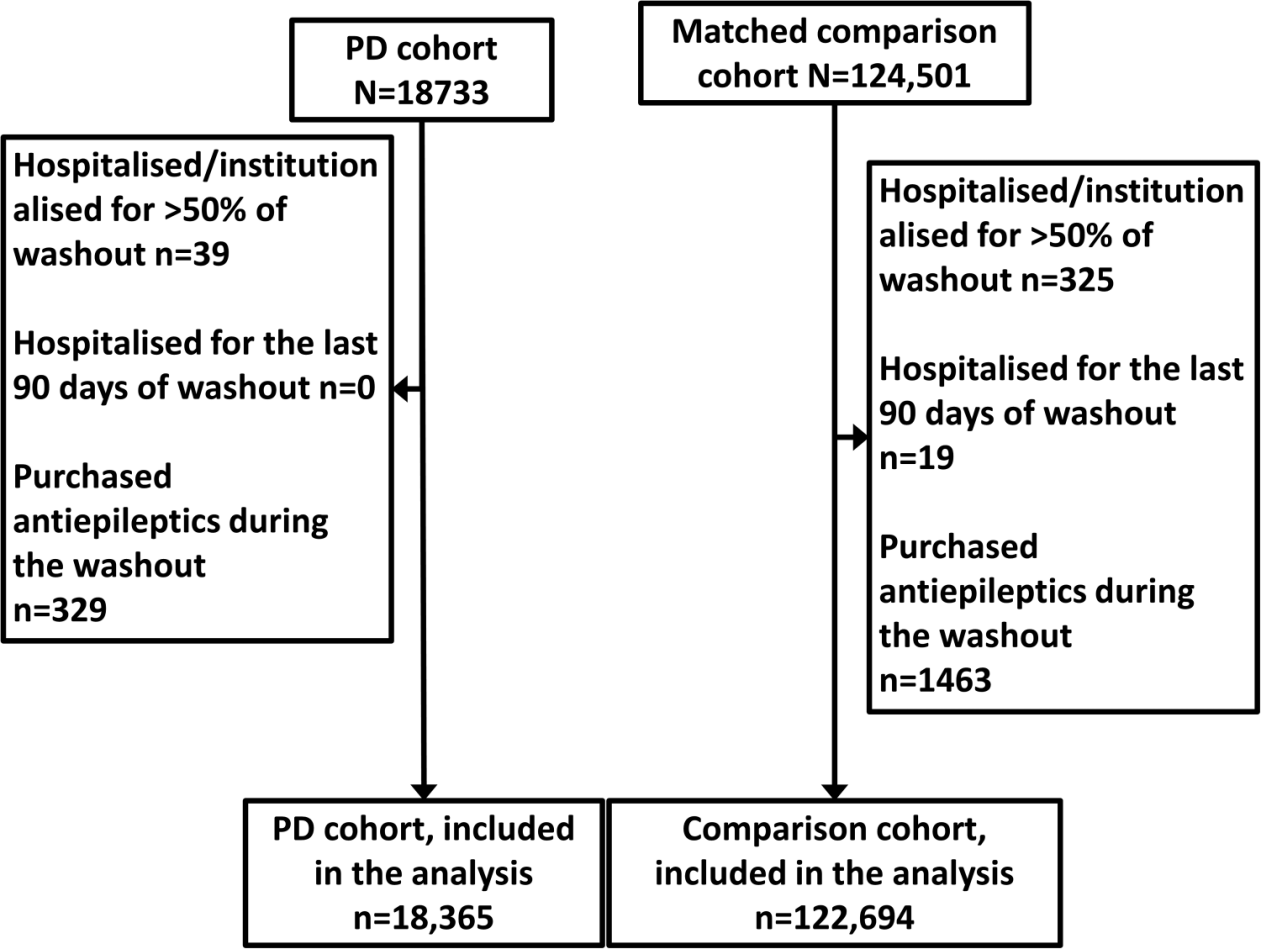
Formation of the study population.

The derivation of study population is described in Figure 1. The maximum follow-up time was from 10 years before to 10 years after the index date. Prevalent antiepileptic users were excluded with one-year washout prior to the follow-up. Year 1995 was used as the washout for those with index date in 2001–2005 or before and follow-up of these persons began in 1996. In addition to prevalent users, we excluded persons who were hospitalized for more than half of the washout or hospitalized for the last 3 months of the washout, as drugs administered in these settings were not recorded in the utilized data sources.

The follow-up ended on antiepileptic initiation, end of the follow-up period, death, or the end of the register linkage (31.12.2019). In addition, if a comparison person was diagnosed with PD, the follow-up ended on the diagnosis date if this occurred earlier than the other reasons for censoring.

Incidence of antiepileptic initiation per 100 person-years was measured in 6-month time windows. If a person spent more than 120 days in a hospital during a single 6-month interval, they were excluded from that time window.

### Antiepileptic use

Antiepileptic use in 1995–2019 was identified from the Prescription register, which covers reimbursed medication. Antiepileptic was defined as any medication with the Anatomical Therapeutic Chemical (ATC) classification code N03A. In addition to any antiepileptic use, we assessed the initiation of gabapentinoids (pregabalin and gabapentin, ATC codes N03AX12 and N03AX16, respectively), other antiepileptics (ATC N03A other than N03AX12 and N03AX16), and clonazepam (ATC code N03AE01).

### Covariates

Information of the following comorbidities was obtained from the Care register for Health Care and Special Reimbursement register: epilepsy, schizophrenia, bipolar disorder or mania, other mood disorder, asthma, or chronic obstructive pulmonary disease (COPD), cardiovascular disease (CVD), stroke, diabetes, cancer, head injuries and substance abuse. In addition, Prescription register was used to identify the use of following medications: antidepressants, antipsychotic medication, benzodiazepines and related drugs (BZDR), opioids, paracetamol or non-steroidal anti-inflammatory drugs (NSAIDs). In addition to any antidepressant, tricyclic antidepressants, as well as duloxetine and venlafaxine were assessed separately as they may be used for neuropathic pain.

The time windows, data sources and codes used to identify the covariate information are listed in Supplemental Table 1. Occupational social class was used as a proxy for socioeconomic position using the categorization of Statistics Finland.

### Statistical analysis

The differences in age, sex, occupational social class, comorbidities, and use of medications between antiepileptic initiators and non-initiators were assessed separately for persons with and without PD. In addition, the differences in these characteristics and time since index date were assessed between antiepileptic initiators with and without PD. T-test was used for continuous variables with normal distribution, Mann-Whitney U-test for continuous variables with skewed distribution and chi-squared test for categorical variables. For the comparison between initiators and non-initiators, covariates were measured before the follow-up period. For the comparisons between initiators with and without PD, the covariates were measured until the initiation date.

Incidence ratios were compared using Poisson regression to obtain incidence rate ratios. The matching characteristics (age, sex, and region) were accounted by using clustered sandwich estimator for variance estimation Statistical analyses were done using STATA MP 17.0.

## Results

The study population consisted of 18,365 people with PD and 122,694 people without PD (Table 1, Supplemental Table 2). The mean age on index date was 70.7 years and 55.8% of the study population were men. In both cohorts, the proportion of women was higher among initiators than non-initiators. Although there were differences in the distribution of age and occupational social class between initiators and non-initiators, they were small and had no consistent pattern. In general, the prevalence of comorbidities and use of analgesic and psychotropic medication was higher among antiepileptic initiators than non-initiators in both cohorts. Epilepsy was quite rare (prevalence <1%) in initiators and non-initiators, with and without PD before the follow-up.

**Table 1.** The characteristics of initiators and non-initiators with and without PD. Initiators were identified with one-year washout before the follow-up.

	PD, N = 18,365			No PD, N = 122,694		
	Initiators n (%)	Non-initiators n (%)	p	Initiators n (%)	Non-initiators n (%)	p
	5386 (29.3)	12979 (70.7)		18643 (15.2)	104051 (84.8)	
Age at the index date, mean (95% CI)	69.6 (69.3–69.8)	71.6 (71.4–71.7)	<0.001	71.2 (71.1–71.3)	70.4 (70.4–70.5)	<0.001
Sex			<0.001			<0.001
Women	2726 (50.6)	5442 (41.9)		9262 (49.7)	44881 (43.1)	
Men	2660 (49.4)	7537 (58.1)		9381 (50.3)	59170 (56.9)	
Highest occupational social class			<0.001			<0.001
Lower-level employees	1442 (26.8)	2912 (22.4)		4816 (25.8)	23562 (22.6)	
Self-employed	1343 (24.9)	3555 (27.4)		4599 (24.7)	27287 (26.2)	
Manual workers	1315 (24.4)	3380 (26.0)		5279 (28.3)	29028 (27.9)	
Upper-level employees	1092 (20.3)	2500 (19.3)		3089 (16.6)	18227 (17.5)	
Pensioners	150 (2.8)	533 (4.1)		723 (3.9)	3789 (3.6)	
Others	44 (0.82)	99 (0.76)		137 (0.73)	2158 (2.1)	
Comorbidities before follow-up period						
Asthma or chronic obstructive pulmonary disease	322 (6.0)	588 (4.5)	<0.001	1259 (6.8)	4721 (4.5)	<0.001
Cancer	46 (0.85)	149 (1.2)	0.077	202 (1.1)	897 (0.86)	0.003
Cardiovascular disease	1514 (28.1)	3855 (29.7)	0.031	6060 (32.5)	28518 (27.4)	<0.001
Diabetes	284 (5.3)	634 (4.9)	0.272	1231 (6.6)	4462 (4.3)	<0.001
Stroke	88 (1.6)	181 (1.4)	0.219	454 (2.4)	1730 (1.7)	<0.001
Epilepsy	38 (0.71)	31 (0.24)	<0.001	131 (0.70)	256 (0.25)	<0.001
Head injury	122 (2.3)	173 (1.3)	<0.001	495 (2.7)	1866 (1.8)	<0.001
Schizophrenia	58 (1.1)	92 (0.7)	0.012	178 (0.95)	645 (0.6)	<0.001
Bipolar disorder	52 (0.97)	32 (0.25)	<0.001	111 (0.60)	211 (0.20)	<0.001
Mood disorders other than bipolar	183 (3.4)	201 (1.6)	<0.001	580 (3.1)	1314 (1.3)	<0.001
Substance abuse	97 (1.8)	120 (0.92)	<0.001	489 (2.6)	1456 (1.4)	<0.001
Medication use during washout period						
Non-steroidal anti-inflammatory drugs	1510 (28.0)	2798 (21.6)	<0.001	6129 (32.9)	21341 (20.5)	<0.001
Paracetamol	31 (0.58)	29 (0.22)	<0.001	108 (0.58)	212 (0.20)	<0.001
Opioids	156 (2.9)	159 (1.2)	<0.001	735 (3.9)	1312 (1.3)	<0.001
Any antidepressant	544 (10.1)	574 (4.4)	<0.001	1655 (8.9)	3924 (3.8)	<0.001
Duloxetine/venlafaxine	17 (0.3)	14 (0.11)	0.002	49 (0.26)	95 (0.09)	<0.001
Tricyclic antidepressants	178 (3.3)	187 (1.4)	<0.001	587 (3.2)	1208 (1.2)	<0.001
Antipsychotic medication	228 (4.2)	322 (2.5)	<0.001	501 (2.7)	1548 (1.5)	<0.001
Benzodiazepines or related medication	929 (17.3)	1363 (10.5)	<0.001	3357 (18.0)	9584 (9.2)	<0.001

**Figure 2.**
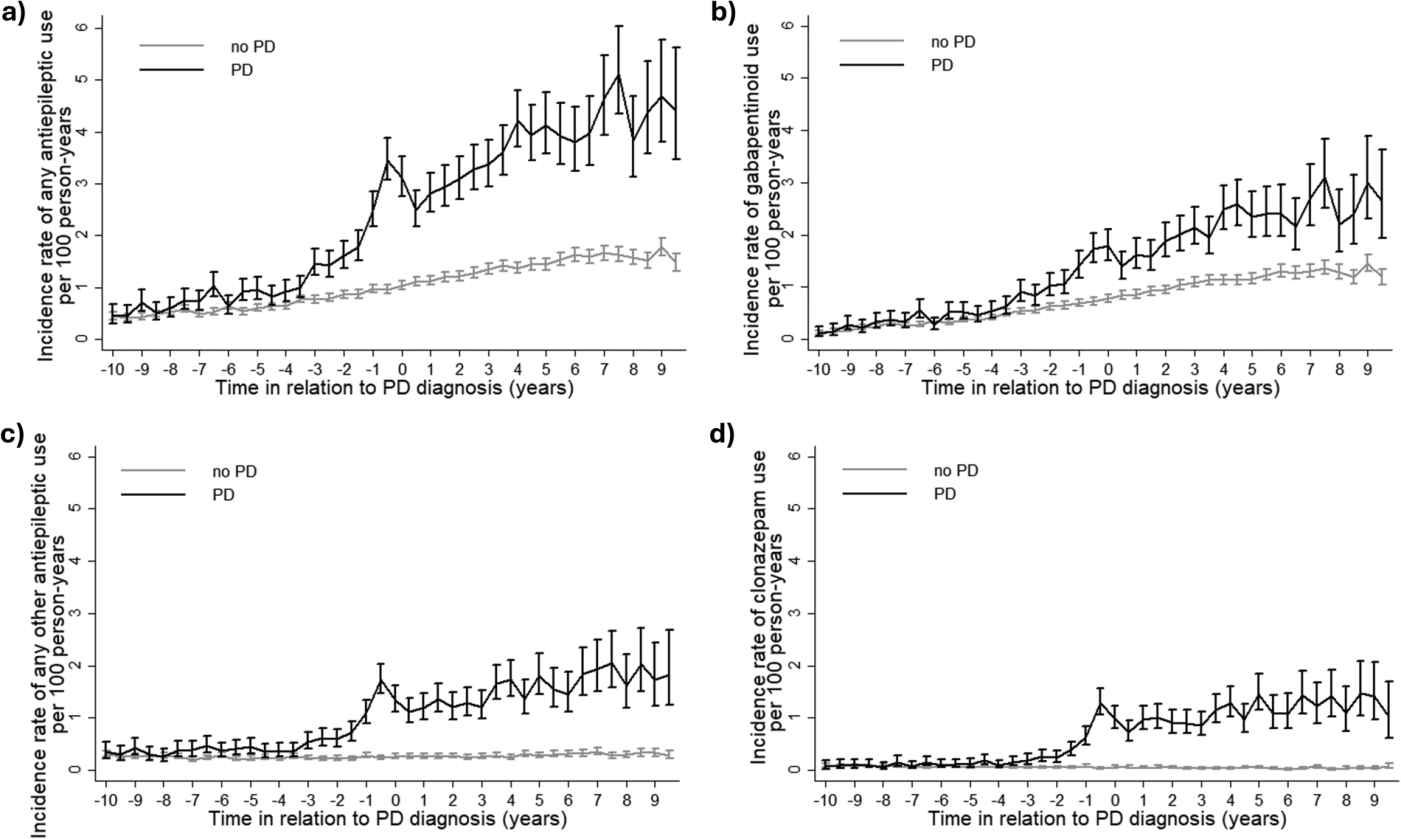
Incidence of antiepileptic use in relation to the time since index date (PD diagnosis, year 0) in persons with and without PD for (a) any antiepileptic, (b) gabapentinoids, (c) antiepileptic other than gabapentinoids, (d) clonazepam. Data are shown as incidence rate per hundred person-years with 95% confidence intervals.

Altogether 29.3% of persons with PD and 15.2% without PD initiated any antiepileptics during the follow-up. The initiation rate of any antiepileptic increased throughout the follow-up in both cohorts, but the increase in persons with PD was steeper, and the difference to the comparison cohort began to emerge approximately 3 years before the index date (Figure 2(a)). The highest incidence rate ratio was observed 0.5 years before the index date (3.56, with confidence interval of 3.09–4.10, Supplemental Table 3). Similar pattern was observed for gabapentinoid use (Figure 2(b)). For other antiepileptics and clonazepam, the incidence was stable in the comparison cohort throughout the follow-up while the increase in the PD cohort began approximately 3 years before the index date, spiked just before the index date and continued on a higher level than in the comparison cohort until the end of the follow-up (Figure 2(c, d)).

There were no clinically meaningful differences in the age at antiepileptic initiation, sex, or time since index date between initiators with and without PD (Table 2). Psychiatric comorbidities were more common among initiators with PD, although the differences were small. There were no large differences in the prevalence of epilepsy and head injuries between the groups while other somatic comorbidities and substance abuse were more common in initiators without PD. The use of psychotropic medications was more common among initiators with PD, while use of paracetamol and opioids was slightly more common among initiators without PD.

**Table 2.** The characteristics of initiators of any antiepileptic with and without PD.

	Initiators with PD, n (%) n = 5386	Initiators without PD, n (%) n = 18643	p
Age at initiation, mean (95% CI)	71.2 (70.9–71.4)	72.5 (72.4–72.7)	<0.001
Sex			0.228
Women	2726 (50.6)	9262 (49.7)	
Men	2660 (49.4)	9381 (50.3)	
Time since diagnosis at antiepileptic initiation in years, median (IQR)	1.6 (−1.1–4.9)	1.3 (−2.3–5.3)	0.016
Highest occupational social class			<0.001
Lower-level employees	1442 (26.8)	4816 (25.8)	
Self-employed	1343 (24.9)	4599 (24.7)	
Manual workers	1315 (24.4)	5279 (28.3)	
Upper-level employees	1092 (20.3)	3089 (16.6)	
Pensioners	150 (2.8)	723 (3.9)	
Others	44 (0.82)	137 (0.73)	
Comorbidities before initiation			
Asthma or chronic obstructive pulmonary disease	497 (9.2)	2112 (11.3)	0.001
Cancer	170 (3.2)	865 (4.6)	<0.001
Cardiovascular disease	2194 (40.7)	8562 (45.9)	<0.001
Diabetes	868 (16.1)	3607 (19.4)	<0.001
Stroke	507 (9.4)	2432 (13.1)	<0.001
Epilepsy	197 (3.7)	781 (4.2)	0.082
Head injury	474 (8.8)	1785 (9.6)	0.086
Schizophrenia	150 (2.8)	356 (1.9)	<0.001
Bipolar disorder	100 (1.9)	249 (1.3)	0.005
Mood disorders other than bipolar	593 (11.0)	1393 (7.5)	<0.001
Substance abuse	232 (4.3)	1107 (5.9)	<0.001
Medication use during washout period			
Non-steroidal anti-inflammatory drugs	2448 (45.5)	8745 (46.9)	0.059
Paracetamol	1851 (34.4)	6864 (36.8)	0.001
Opioids	1515 (28.1)	6252 (33.5)	<0.001
Any antidepressant	1618 (30.0)	3907 (21.0)	<0.001
Duloxetine or venlafaxine	215 (4.0)	542 (2.90)	<0.001
Tricyclic antidepressants	222 (4.1)	825 (4.4)	0.337
Antipsychotic medication	717 (13.3)	1349 (7.2)	<0.001
Benzodiazepines or benzodiazepine-related medication	1791 (33.3)	5878 (31.5)	0.017

Altogether 57.1% of antiepileptic initiators with PD and 72.3% without PD initiated with gabapentinoids, and gabapentinoids were more commonly initiated than other antiepileptics. The most commonly initiated antiepileptic in both cohorts was pregabalin (41.0% and 55.3% of antiepileptic initiations in persons with and without PD, respectively), followed by clonazepam (26.7%), gabapentin (16.1%) and valproate (6.0%) in persons with PD (Supplemental Table 4). In persons without PD, gabapentin was initiated by 16.9%, carbamazepine by 7.9% and valproate and clonazepam by approximately 6% each. A similar proportion of persons with PD (n = 33, 0.60% of initiators) and without PD (n = 148, 0.79%) initiated with more than one antiepileptic.

Gabapentinoid initiators with PD were on average two years older at the antiepileptic initiation than those with PD who initiated with other antiepileptics (Table 3). Most of the initiators of gabapentinoids were women, compared to initiators of other antiepileptics, of which majority were men. Gabapentinoid initiators with PD had higher prevalence of CVD, asthma or COPD and diabetes, but lower prevalence of head injury, stroke, or mood disorders, compared to initiators of other antiepileptics. The initiators of other antiepileptics had much higher prevalence of epilepsy (7.9%) compared to the initiators of gabapentinoids (0.45%). Initiators of gabapentinoids with PD had higher use of analgesics compared to initiators of other antiepileptics.

**Table 3.** Characteristics of initiators with PD of gabapentinoids versus initiators with PD of other antiepileptics. Persons who initiated both a gabapentinoid and other antiepileptic (*n* *=* 17) were categorized as initiators for gabapentinoids in this table.

	Gabapentinoid initiators with PD, n (%)	Initiators of other antiepileptics with PD, n (%)	p
	3096 (57.5)	2290 (42.5)	
Age at initiation, mean (95% CI)	72.1 (71.8–72.4)	69.9 (69.5–70.3)	<0.001
Sex			<0.001
Women	1693 (54.7)	1033 (45.1)	
Men	1403 (45.3)	1257 (54.9)	
Years since index date at initiation, median (IQR)	1.8 (−0.96–5.0)	1.3 (−1.3–4.8)	0.001
Highest occupational social class			<0.001
Lower-level employees	899 (29.0)	543 (23.7)	
Self-employed	764 (24.7)	579 (25.3)	
Manual workers	742 (24.0)	573 (25.0)	
Upper-level employees	603 (19.5)	489 (21.4)	
Pensioners	64 (2.1)	86 (3.7)	
Others	24 (0.78)	20 (0.87)	
Comorbidities before initiation			
Asthma or chronic obstructive pulmonary disease	330 (10.7)	167 (7.3)	<0.001
Cancer	91 (2.9)	79 (3.5)	0.289
Cardiovascular disease	1324 (42.8)	870 (38.0)	<0.001
Diabetes	560 (18.1)	308 (13.5)	<0.001
Stroke	244 (7.9)	263 (11.5)	<0.001
Epilepsy	14 (0.45)	183 (7.9)	<0.001
Head injury	265 (8.6)	209 (9.2)	0.468
Schizophrenia	43 (1.4)	107 (4.7)	<0.001
Bipolar disorder	26 (0.84)	74 (3.2)	<0.001
Mood disorders other than bipolar	302 (9.8)	291 (12.7)	0.001
Substance abuse	131 (4.2)	101 (4.4)	0.749
Medication use one year before antiepileptic initiation			
Non-steroidal anti-inflammatory drugs	1739 (56.2)	709 (31.0)	<0.001
Paracetamol	1376 (44.4)	475 (20.7)	<0.001
Opioids	1216 (39.3)	299 (13.1)	<0.001
Any antidepressant	892 (28.8)	726 (31.7)	0.022
Duloxetine or venlafaxine	132 (4.3)	83 (3.6)	0.236
Tricyclic antidepressants	134 (4.3)	88 (3.8)	0.376
Antipsychotic medication	297 (9.6)	420 (18.3)	<0.001
Benzodiazepines or benzodiazepine-related medication	1055 (34.1)	736 (32.1)	0.136

In comparison to gabapentinoid initiators without PD, gabapentinoid initiators with PD were approximately one year younger at initiation, had lower prevalence of somatic comorbidities such as CVD or diabetes, but slightly higher prevalence of mood and bipolar disorders (Supplemental Table 5). Initiators of gabapentinoids with PD also had higher prevalence of use of all studied medications except for opioids. In the comparison of the initiators of other antiepileptics with and without PD, there was no significant age difference. Initiators with PD initiated antiepileptics on average 2 years later than initiators without PD, and had fewer comorbidities except for schizophrenia and mood disorders other than bipolar, such as mania and depression. Use of antidepressants and paracetamol were also more common among initiators with PD.

## Discussion

In our longitudinal study the incidence of any antiepileptic initiation was higher in persons with PD, and the difference became evident already 3 years before the diagnosis in comparison to a matched cohort of persons without PD. The difference was observed for both gabapentinoids and other antiepileptics. Clonazepam was the most commonly initiated non-gabapentinoid antiepileptic in persons with PD. There was a peak in initiations of any antiepileptics before the index date in persons with PD, and the initiation rates remained above the comparison cohort until the end of the follow-up.

In the PD population the emerging difference to the comparison cohort 3 years before the index date may reflect non-motor symptoms which can start before or concurrently with motor symptoms.^9,10,17^ This increase before the diagnosis was observed for both gabapentinoids and other antiepileptics, and therefore there are likely several reasons, or symptoms, that contribute to increased incidence in persons with PD including neuropathic pain, anxiety, restless legs and postural or kinetic tremor initially classified as essential tremor. Symptoms such as anxiety had higher prevalences in the prediagnostic phase of PD, suggesting their contribution.^9^

Gabapentinoids were more commonly initiated among persons with PD throughout the study period, which likely reflects the higher prevalence of pain in persons with PD.^11^ Pain is a possible prodromal symptom of PD, as around 20% of people with undiagnosed PD have symptoms of pain starting from few years before the diagnosis.^18^ We have previously shown that the incidence of muscle relaxants also began to increase 3 years before the diagnosis in persons with PD,^19^ suggesting the increase in musculoskeletal symptoms, requiring pharmacotherapy in this time window.^9^ The use of analgesics, opioids, tricyclic antidepressants, duloxetine, and venlafaxine was more common among the gabapentinoid initiators than among those who initiated with other antiepileptics, regardless of the presence of PD, which is in line with neuropathic pain being an indication for gabapentinoids. In addition, anxiety may also partly explain the initiation of pregabalin.^20^

During the follow-up time, the rate of gabapentinoid initiations rose steadily in both cohorts, which could reflect age related increase in neuropathic pain due to for example diabetes and postherpetic neuralgia,^21^ and due to increase of gabapentinoid use for several indications in Finland.^22^ Pregabalin was authorized by the European Medicines Agency for use in 2004, which was in the middle of the study. In addition, disease progress in PD can lead to the worsening of symptoms, leading to alterations in pharmacotherapy. There was a sex difference in antiepileptic initiation in PD, as gabapentinoid initiators were more likely to be women, while a larger proportion of initiators of other antiepileptics were men. Neuropathic pain is more common in women,^21^ and a previous study reported that female gender was the primary predictor of pain in PD.^7^

The initiation of other antiepileptics was significantly more common in the population with PD than in the comparison cohort. This difference is not explained by the prevalence of stroke, epilepsy, or substance abuse in the PD cohort, as these conditions were more common in comparison cohort. The incidence of other antiepileptic use rose 4 to 3 years before the date of diagnosis and then remained on the same level for the rest of the follow-up period. Thus, the initiation of other antiepileptics seems more connected to both the prodromal stage and progression of PD. In addition, epilepsy and bipolar disorder were more common among those who initiated with other antiepileptics than gabapentinoids, which is in line with the indications of these medications. Interestingly, both bipolar disorder and epilepsy have been associated with higher risk of PD.^10,12^

Clonazepam amounted to a quarter of all initiations of antiepileptics for people with PD, while for the matched cohort the rate was only about 6%. Clonazepam is used for treatment of severe epilepsy in combination with other antiepileptics. It can also be used for rapid eye movement sleep behavior disorder (RBD) in PD,^23^ and clonazepam is mentioned as a treatment option for RBD in the Finnish Current Care Guideline for PD.^24^ As a benzodiazepine, clonazepam has possible usage for anxiety, which is one of the common non-motor symptoms of PD.^25^ Anxiety and RBD are strongly linked to PD, which could explain the low incidence of clonazepam use in the comparison group. Dystonia is one of possible early motor sign of PD^26^ and this could also partially explain the higher use of clonazepam in the PD group given the off-label use of the drug for dystonic symptoms.^27^

Before prescribing antiepileptics careful consideration of benefits and harms is needed as the use of antiepileptics is related to higher risk of adverse effects and events, like falls and related injuries like hip fractures and traumatic brain injuries in older population.^28^ Possible causes related to fall risk are for example dizziness, sedation, and hyponatremia caused by carbamazepine, oxcarbazepine and valproate. In addition, carbamazepine and oxcarbazepine have interactions with other medications via the cytochrome P450 enzyme (CYP3A4).^29^ These medication related adverse effects and events are especially important in persons with PD who have a higher risk for falls already before the diagnosis.^30^

The strengths of this study arise from the applied data sources. We were able to conduct a nationwide drug utilization study with purchased prescriptions and comorbidity data. In addition, we were able to assess the incidence over a 20-year period, which provides important information on changes in initiation rates in relation to PD diagnosis. However, there are some limitations as we had no information on severity of PD, or the indication of the initiated antiepileptics. In addition, the prescription register includes information on reimbursed prescription drugs, meaning that the prevalence of NSAIDs and paracetamol, that are commonly purchased as over-the-counter is underestimated in our study.

In conclusion, persons with PD were more likely to initiate antiepileptics already 3 years before PD diagnosis in comparison to a matched cohort without PD, and the difference remained after the diagnosis until the end of the study. Gabapentinoids constituted a larger proportion of initiated antiepileptics in persons without PD while other antiepileptics were more common in persons with PD. The increased incidence of these medications likely reflects treatment of PD-associated symptoms such as neuropathic pain and anxiety and underlines the clinical importance of early non-motor PD symptoms and their treatment needs. It is important to assess harms and benefits of antiepileptics before prescribing and review medication regularly during the use to avoid adverse effects and events related to antiepileptic use, including falls and injuries.

## Supplemental Material

sj-docx-1-pkn-10.1177_1877718X251343079 - Supplemental material for Incidence of antiepileptic drug use in Parkinson's disease Supplemental material, sj-docx-1-pkn-10.1177_1877718X251343079 for Incidence of antiepileptic drug use in Parkinson's disease by Santeri Tuominen, Miia Tiihonen, Anne Paakinaho, Marjaana Koponen, Valtteri Kaasinen, Sirpa Hartikainen and Anna-Maija Tolppanen in Journal of Parkinson's Disease
