# Supplementary material for: Incidence of antiepileptic drug use in Parkinson's disease
Source: J Parkinsons Dis. 2025 May 23:1877718X251343079. Online ahead of print. doi: 10.1177/1877718X251343079 (PMC13347489; doi:10.1177/1877718X251343079)
Supplement: Supplementary file 1 [file sj-docx-1-pkn-10.1177_1877718X251343079.docx]

**Supplemental Material**

**Incidence of antiepileptic drug use in Parkinson’s disease**

**Supplemental Table 1.** Variables and comorbidities used in the study.

| Variable | Data sources & coding | Years |
| --- | --- | --- |
| Occupational social class | 1: Self-employed  2: Upper-level employees with administrative, managerial, professional, and related occupations  3: Lower-level employees with administrative and clerical occupations  4: Manual workers  5: Pensioners  6: Others (Students, long-term unemployed, other positions not elsewhere classified, socioeconomic status unknown)  ref: https://www.stat.fi/fi/luokitukset/sosioekon_asema/ | before the follow-up |
| Time since index date | (Years, negative values=before index date, positive values=after the index date) | initiation date |
| Schizophrenia | Care Register for Health Care  ICD 9: 295, 297, 298  ICD-10: F20-F29 | From 1987 to the beginning of follow-up/initiation date |
| Bipolar disorder or mania | Care Register for Health Care  ICD 9: 2962, 2963, 2964, 2967  ICD-10: F30, F31 | From 1987 to the beginning of follow-up/initiation date |
| Any mood disorder (other than bipolar) | Care Register for Health Care  ICD-9: 2961, 2968A, 3004A, 3011D  ICD-10: F32-F39 | From 1987 to the beginning of follow-up/initiation date |
| Epilepsy | Special reimbursement register code 111 | From 1987 to the beginning of follow-up/initiation date |
| Asthma/chronic obstructive pulmonary disease | Special reimbursement register code 203 | From 1987 to the beginning of follow-up/initiation date |
| Cardiovascular disease | Special reimbursement register code 201, 205, 206, 207, 213, 280 | From 1987 to the beginning of follow-up/initiation date |
| Stroke | Care register for health care  ICD-9: 430-432,4360,4330A,4331A,4339A,4340A,4341A,4349A,4380A  ICD-10 I60-I64, I69 | From 1987 to the beginning of follow-up/initiation date |
| Diabetes | Prescription register: ATC code A10 excluding A10BX01(guar gum)  Special reimbursement register code 103 | From 1995 to the beginning of follow-up/initiation date  From 1987 to the beginning of follow-up/initiation date |
| Cancer | Cancer register: International Agency for Research on Cancer (CRG: Collaborative Research Group) (IARC Tools) code C* | Within two years before the beginning of follow-up/initiation date |
| Head injuries | ICD-10: S00-S09  ICD-9: 800–803,830, 8480, 850–854, 870–873, 918, 920, 921, 9250, 9251, 950, 951, 9590 | From 1987 to the beginning of follow-up/initiation date |
| Substance abuse | Prescription register: ATC codes N07BB, N07BC  Care register for health care  Reason for admission: (33,71,72,73,74,75)  ICD-10 F1*, K860, K70, G621, G312, G721, I426, K292, R78  ICD-9 291,292,303,304,305,3575, 3594A, 4255A,5353A,5770D-F,5771C,5710A,5711A, 5712A,5713X | from 1995 to the beginning of follow-up/initiation date  From 1987 to the beginning of follow-up/initiation date |
| Any antidepressant use | Prescription register: ATC code N06A | washout/One year before the initiation date |
| Tricyclic antidepressant use | Prescription register: ATC code N06AA | washout/One year before the initiation date |
| Duloxetine/venlafaxine | Prescription register: ATC code N06AX21, N06AX16 | washout/One year before the initiation date |
| Antipsychotic use | Prescription register: ATC code N05A  (Excluding N05AN01 and N05AB04) | washout/One year before the initiation date |
| Benzodiazepines and related medication | Prescription register: ATC code N05BA, N05CD, N05CF | washout/One year before the initiation date |
| Opioid use | Prescription register: ATC code N02A | washout/One year before the initiation date |
| Paracetamol use | Prescription register: ATC code N02BE01, N02AJ01, N02AJ06, N02AJ17, N02AJ13, N02BE51, N02BE71 | washout/One year before the initiation date |
| Non-steroidal anti-inflammatory drug use | Prescription register: ATC code M01A, N02AJ08, N02AJ14 | washout/One year before the initiation date |

**Supplemental Table 2.** Hospital districts of initiators and non-initiators with and without PD. Initiators were identified with one-year washout before the follow-up.

|  | **PD, N=18,365** | | | | **No PD, N=122,694** | | |
| --- | --- | --- | --- | --- | --- | --- | --- |
|  | **Initiators n (%)** | **Non-initiators n (%)** | **p** | **Initiators n (%)** | | **Non-initiators n (%)** | **p** |
|  | 5386 (29.3) | 12979 (70.7) |  | 18643 (15.2) | | 104051 (84.8) |  |
| **Hospital district on index date** | |  | <0.001 |  | |  | <0.001 |
| Helsinki and Uusimaa | 1249 (23.2) | 2808 (21.6) |  | 4055 (21.8) | | 23313 (22.4) |  |
| Varsinais-Suomi | 591 (11.0) | 1037 (8.0) |  | 1904 (10.2) | | 8928 (8.6) |  |
| Satakunta | 215 (4.0) | 577 (4.4) |  | 811 (4.4) | | 4466 (4.3) |  |
| Kanta-Häme | 133 (2.5) | 451 (3.5) |  | 576 (3.1) | | 3344 (3.2) |  |
| Pirkanmaa | 397 (7.4) | 1149 (8.9) |  | 1395 (7.5) | | 8863 (8.5) |  |
| Päijät-Häme | 241 (4.5) | 564 (4.3) |  | 821 (4.4) | | 4626 (4.4) |  |
| Kymenlaakso | 179 (3.3) | 475 (3.7) |  | 607 (3.3) | | 3766 (3.6) |  |
| Etelä-Karjala | 110 (2.0) | 423 (3.3) |  | 448 (2.4) | | 3110 (3.0) |  |
| Etelä-Savo | 146 (2.7) | 282 (2.2) |  | 447 (2.4) | | 2388 (2.3) |  |
| Itä-Savo | 77 (1.4) | 230 (1.8) |  | 248 (1.3) | | 1795 (1.7) |  |
| Pohjois-Karjala | 262 (4.9) | 639 (4.9) |  | 917 (4.9) | | 5155 (5.0) |  |
| Pohjois-Savo | 280 (5.2) | 683 (5.3) |  | 920 (4.9) | | 5443 (5.2) |  |
| Keski-Suomi | 278 (5.2) | 671 (5.2) |  | 1011 (5.4) | | 5358 (5.1) |  |
| Etelä-Pohjanmaa | 229 (4.3) | 699 (5.4) |  | 920 (4.9) | | 5234 (5.0) |  |
| Vaasa | 143 (2.7) | 433 (3.3) |  | 435 (2.3) | | 3405 (3.3) |  |
| Keski-Pohjanmaa | 87 (1.6) | 218 (1.7) |  | 329 (1.8) | | 1702 (1.6) |  |
| Pohjois-Pohjanmaa | 423 (7.9) | 921 (7.1) |  | 1529 (8.2) | | 7292 (7.0) |  |
| Kainuu | 105 (1.9) | 181 (1.4) |  | 374 (2.0) | | 1508 (1.4) |  |
| Länsi-Pohja | 78 (1.4) | 160 (1.2) |  | 273 (1.5) | | 1325 (1.3) |  |
| Lappi | 142 (2.6) | 300 (2.3) |  | 587 (3.1) | | 2373 (2.3) |  |
| Ahvenanmaa | 16 (0.3) | 54 (0.4) |  | 36 (0.2) | | 454 (0.4) |  |
| Unknown | 5 (0.1) | 24 (0.2) |  | 0 (0.0) | | 203 (0.2) |  |

**Supplemental Table 3.** Incidence rate ratio of antiepileptic initiation between the population with PD and population without PD. Time denotes the beginning of the specific time window (for example, -10 refers to the six-month time window that begins 10 years before the index date. Index date is year 0.

| Time (years) | Incidence rate ratio | 95% confidence interval |
| --- | --- | --- |
| -10 | 1.03 | 0.67-1.56 |
| -9.5 | 1.08 | 0.73-1.60 |
| -9 | 1.62 | 1.18-2.23 |
| -8.5 | 1.06 | 0.74-1.52 |
| -8 | 1.14 | 0.82-1.57 |
| -7.5 | 1.29 | 0.97-1.73 |
| -7 | 1.50 | 1.12-2.01 |
| -6.5 | 1.96 | 1.53-2.52 |
| -6 | 1.00 | 0.75-1.34 |
| -5.5 | 1.73 | 1.35-2.22 |
| -5 | 1.58 | 1.24-2.01 |
| -4.5 | 1.30 | 1.01-1.67 |
| -4 | 1.44 | 1.13-1.83 |
| -3.5 | 1.28 | 1.02-1.61 |
| -3 | 1.90 | 1.56-2.31 |
| -2.5 | 1.79 | 1.47-2.18 |
| -2 | 1.87 | 1.55-2.26 |
| -1.5 | 2.05 | 1.71-2.46 |
| -1 | 2.55 | 2.18-2.99 |
| -0.5 | 3.56 | 3.09-4.10 |
| 0 | 2.98 | 2.58-3.45 |
| 0.5 | 2.23 | 1.90-2.62 |
| 1 | 2.48 | 2.12-2.89 |
| 1.5 | 2.45 | 2.10-2.85 |
| 2 | 2.50 | 2.14-2.91 |
| 2.5 | 2.54 | 2.18-2.96 |
| 3 | 2.45 | 2.11-2.86 |
| 3.5 | 2.52 | 2.17-2.93 |
| 4 | 3.03 | 2.61-3.51 |
| 4.5 | 2.68 | 2.29-3.14 |
| 5 | 2.81 | 2.39-3.30 |
| 5.5 | 2.50 | 2.11-2.97 |
| 6 | 2.30 | 1.92-2.76 |
| 6.5 | 2.44 | 2.03-2.94 |
| 7 | 2.73 | 2.27-3.28 |
| 7.5 | 3.05 | 2.53-3.67 |
| 8 | 2.37 | 1.90-2.96 |
| 8.5 | 2.81 | 2.25-3.52 |
| 9 | 2.56 | 2.04-3.22 |
| 9.5 | 2.94 | 2.24-3.84 |

**Supplemental Table 4.** Numbers of initiations of specific antiepileptics.

| **PD diagnosis** |  |  | **No PD diagnosis** |  |  |
| --- | --- | --- | --- | --- | --- |
| **Medicine** | **Initiations** | **Percent (%)** | **Medicine** | **Initiations** | **Percent (%)** |
| Pregabalin | 2223 | 41.01 | Pregabalin | 10427 | 55.33 |
| Clonazepam | 1445 | 26.66 | Gabapentin | 3178 | 16.90 |
| Gabapentin | 874 | 16.12 | Carbamazepine | 1476 | 7.85 |
| Valproate | 324 | 5.98 | Valproate | 1144 | 6.08 |
| Carbamazepine | 253 | 4.68 | Clonazepam | 1095 | 5.82 |
| Oxcarbazepine | 134 | 2.47 | Oxcarbazepine | 561 | 2.98 |
| Phenytoin | 51 | 0.94 | Phenytoin | 330 | 1.75 |
| Levetiracetam | 47 | 0.86 | Levetiracetam | 332 | 1.77 |
| Lamotrigine | 37 | 0.68 | Lamotrigine | 129 | 0.69 |
| Topiramate | 16 | 0.30 | Topiramate | 83 | 0.44 |
| Primidone | 13 | 0.24 | Primidone | 29 | 0.15 |
| Zonisamide | <5 | <0.05 | Lacosamide | 16 | 0.09 |
| Phenobarbital | <5 | <0.05 | Phenobarbital | 5 | 0.03 |
| Lacosamide | <5 | <0.05 |  |  |  |
| **Total** | **5421** | **100.00** | **Total** | **18804** | **100.00** |
| With 2 or 3 antiepileptics | 33 | 0.61 | With 2 antiepileptics | 136 | 0.73 |
|  |  |  | With 3 or 4 antiepileptics | 12 | 0.06 |

**Supplemental Table 5.** Characteristics of gabapentinoids and other antiepileptics, stratified by PD.

|  | **Initiators of gabapentinoids** | | | | **Initiators of other antiepileptics** | | |
| --- | --- | --- | --- | --- | --- | --- | --- |
|  | **PD, n (%)** | **No PD, n (%)** |  | **PD, n (%)** | | **No PD, n (%)** |  |
|  | 3096 (57.5) | 13604 (73.0) | **p** | 2307 (42.8) | | 5069 (27.0) | **p** |
| **Age at initiation, mean (95% CI)** | 72.1 (71.8-72.4) | 73.4 (73.2-73.5) | <0.001 | 69.9 (69.5-70.3) | | 70.3 (70.0-70.6) | <0.001 |
| **Sex**  Women  Men | 1693 (54.7)  1403 (45.3) | 7030 (51.7)  6574 (48.3) | 0.002 | 1043 (45.2)  1264 (54.8) | | 2244 (44.3)  2825 (55.7) | 0.451 |
| **Years since index date at initiation, median (IQR)** | 1.8 (-0.96 – 5.0) | 2.0 (-1.3 – 5.7) | 0.005 | 1.3 (-1.3 – 4.8) | | -0.43 (-4.9 – 3.9) | <0.001 |
| **Highest occupational social class** | | | <0.001 |  | |  | <0.001 |
| Lower-level employees | 899 (29.0) | 3654 (26.9) |  | 547 (23.7) | | 1169 (23.1) |  |
| Self-employed | 764 (24.7) | 3374 (24.8) |  | 580 (25.1) | | 1234 (24.3) |  |
| Manual workers | 742 (24.0) | 3809 (28.0) |  | 580 (25.1) | | 1474 (29.1) |  |
| Upper-level employees | 603 (19.5) | 2221 (16.3) |  | 494 (21.4) | | 875 (17.3) |  |
| Pensioners | 64 (2.1) | 450 (3.1) |  | 86 (3.7) | | 275 (5.4) |  |
| Others | 24 (0.78) | 96 (0.71) |  | 20 (0.87) | | 20 (0.87) |  |
| **Comorbidities before initiation** | | |  |  | |  |  |
| Asthma or chronic obstructive pulmonary disease | 330 (10.7) | 1684 (12.4) | 0.008 | 170 (7.4) | | 431 (8.5) | 0.099 |
| Cancer | 91 (2.9) | 645 (4.7) | <0.001 | 79 (3.4) | | 223 (4.4) | 0.050 |
| Cardiovascular disease | 1324 (42.8) | 6443 (47.4) | <0.001 | 874 (37.9) | | 2130 (42.0) | >0.001 |
| Diabetes | 560 (18.1) | 2845 (20.9) | <0.001 | 311 (13.5) | | 767 (15.1) | 0.063 |
| Stroke | 244 (7.9) | 1.313 (9.7) | 0.002 | 265 (11.5) | | 1134 (22.4) | <0.001 |
| Epilepsy | 14 (0.45) | 54 (0.40) | 0.663 | 183 (7.9) | | 734 (14.5) | <0.001 |
| Head injury | 265 (8.6) | 1140 (8.4) | 0.745 | 211 (9.2) | | 647 (12.8) | <0.001 |
| Schizophrenia | 43 (1.4) | 144 (1.1) | 0.115 | 109 (4.7) | | 212 (4.2) | 0.290 |
| Bipolar disorder | 26 (0.84) | 59 (0.43) | 0.004 | 74 (3.2) | | 190 (3.8) | 0.247 |
| Mood disorders other than bipolar | 302 (9.8) | 897 (6.6) | <0.001 | 293 (12.7) | | 498 (9.8) | <0.001 |
| Substance abuse | 131 (4.2) | 641 (4.7) | 0.250 | 103 (4.5) | | 471 (9.3) | <0.001 |
| **Medication use one year before antiepileptic initiation** | | | | |  |  |  |
| Non-steroidal anti-inflammatory drug | 1739 (56.2) | 7114 (52.3) | <0.001 | 718 (31.1) | | 1641 (32.4) | 0.286 |
| Paracetamol | 1376 (44.4) | 5915 (43.5) | 0.329 | 480 (20.8) | | 961 (19.0) | 0.063 |
| Opioids | 1216 (39.3) | 5583 (41.0) | 0.072 | 308 (13.4) | | 678 (13.4) | 0.977 |
| Any antidepressant | 892 (28.8) | 2541 (18.7) | p<0.001 | 735 (31.7) | | 1384 (27.3) | <0.001 |
| Duloxetine or venlafaxine | 132 (4.3) | 386 (2.8) | p<0.001 | 84 (3.6) | | 157 (3.1) | 0.223 |
| Tricyclic antidepressants | 134 (4.3) | 534 (3.9) | p<0.302 | 88 (3.8) | | 294 (5.8) | <0.001 |
| Antipsychotic medication | 297 (9.6) | 518 (3.8) | p<0.001 | 425 (18.4) | | 834 (16.5) | 0.037 |
| Benzodiazepines or related medication | 1055 (34.1) | 4168 (30.6) | p<0.001 | 747 (32.4) | | 1720 (33.9) | 0.190 |
